# Supplementary material for: Histological, Immunohistochemical and Antioxidant Analysis of Skin Wound Healing Influenced by the Topical Application of Brazilian Red Propolis
Source: Antioxidants (Basel). 2022 Nov 4;11(11):2188. doi: 10.3390/antiox11112188 (PMC9686645; doi:10.3390/antiox11112188)
Supplement: Supplementary file 1 [file antioxidants-11-02188-s001.zip › antioxidants-1976421-supplementary.pdf]

Article

Histological, immunohistochemical and antioxidant analysis of skin wound healing influenced by the topical application of brazilian red propolis

Supplementary materials

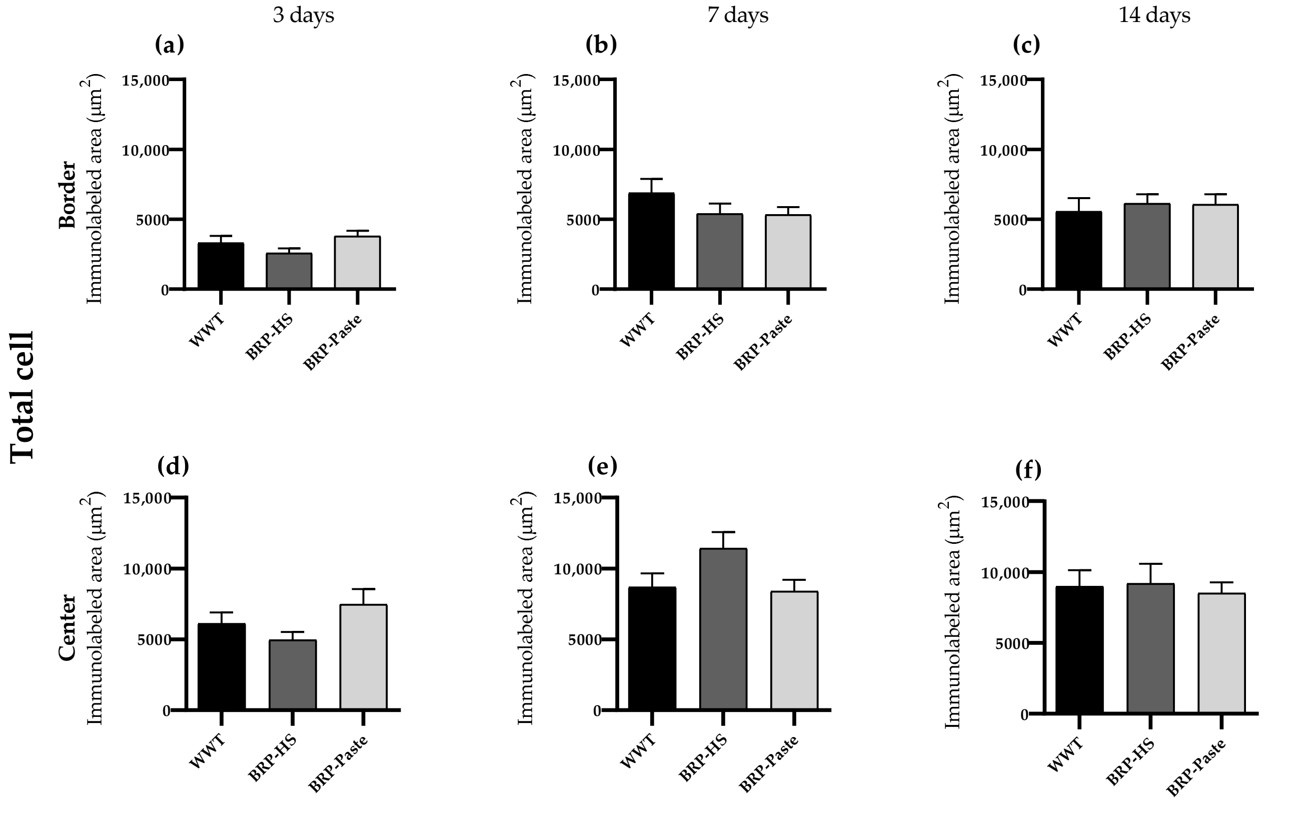

Figure S1. Quantification of total cell in HE stained skin samples (dermis) by area (μm^2) at border (a, b, c) and central (d, e, f) regions of the wounds in each experimental group for the periods of 3 (a, d), 7 (b, e) and 14 (c, f) days of treatment. No statistical difference compared to the WWT group by one-way ANOVA followed by Newman-Keuls (n=6).

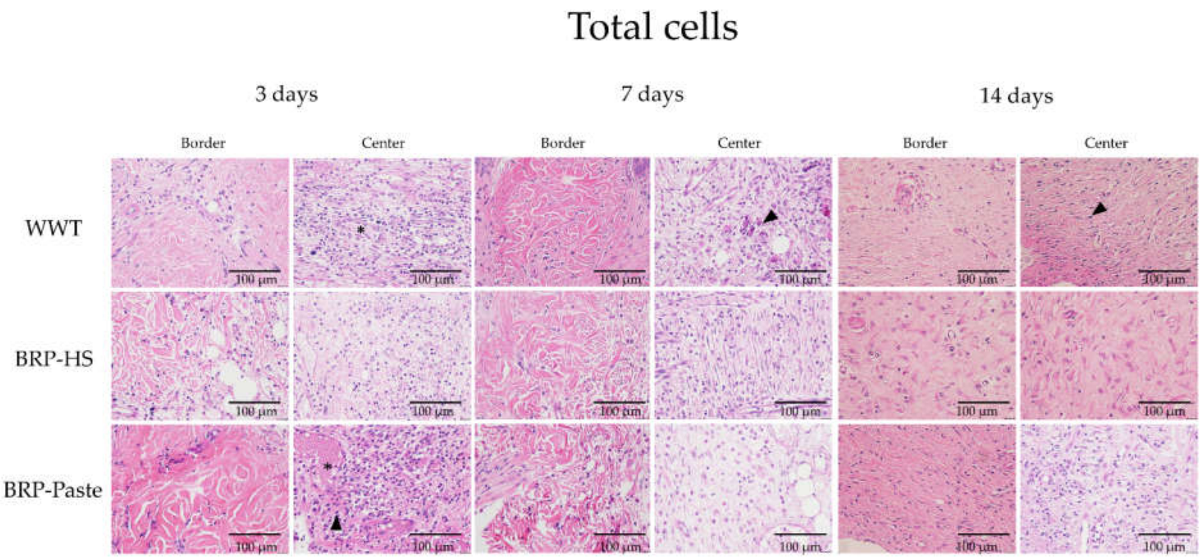

Figure S2. Photomicrographs of the dermis (40x), border and central regions of wounds from each experimental group in 3, 7 and 14 days of treatment for total cell count stained in HE. Bars 100 µm. Arrowheads represent nucleus of inflammatory cells. Asterisks shows blood vesssels.

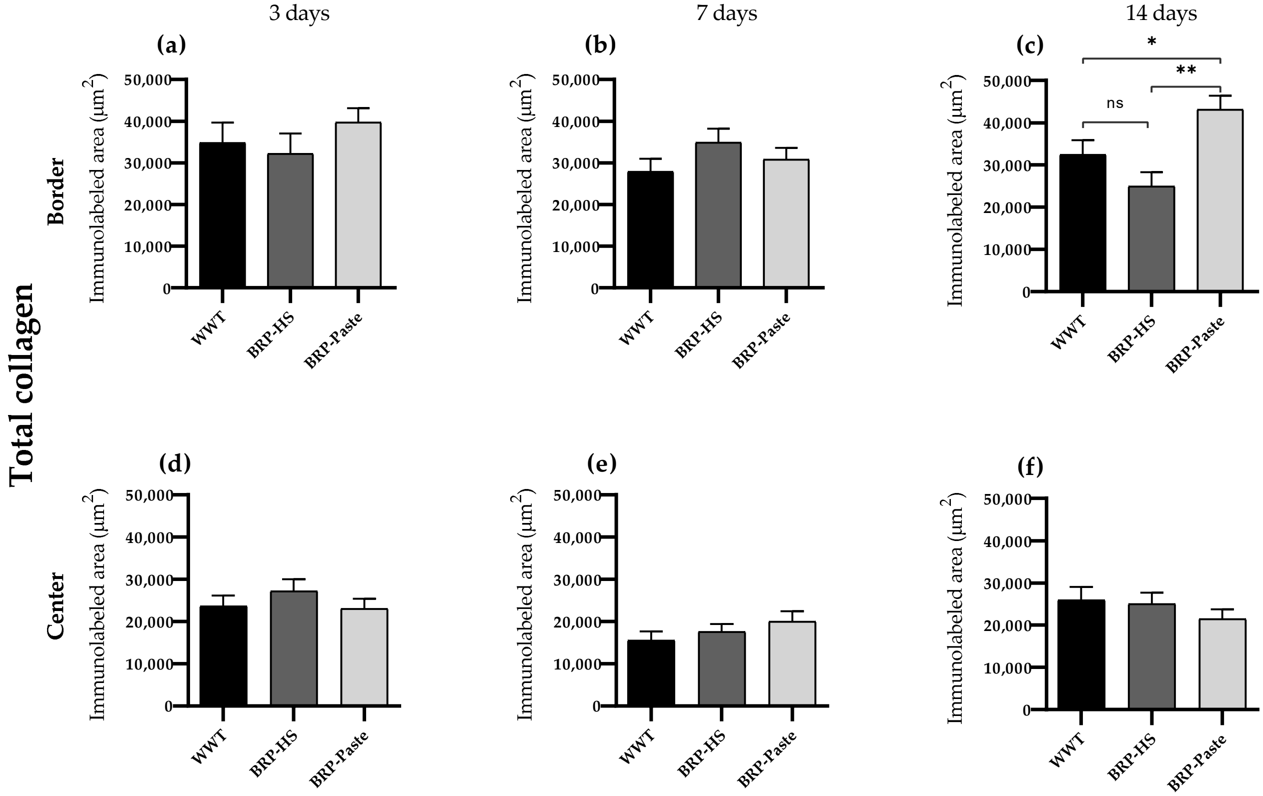

Figure S3. Quantification of total collagen in Mallory's trichrome stained skin samples (dermis) by area (µm^2) at border (a, b, c) and central (d, e, f) regions of the wounds in each experimental group for the periods of 3 (a, d), 7 (b, e) and 14 (c, f) days of treatment. Statistical difference compared to the WWT group was obtained by one-way ANOVA followed by Newman-Keuls test in which $*p < 0.05$ and $**p < 0.01$ (n=6). ns = no significance.

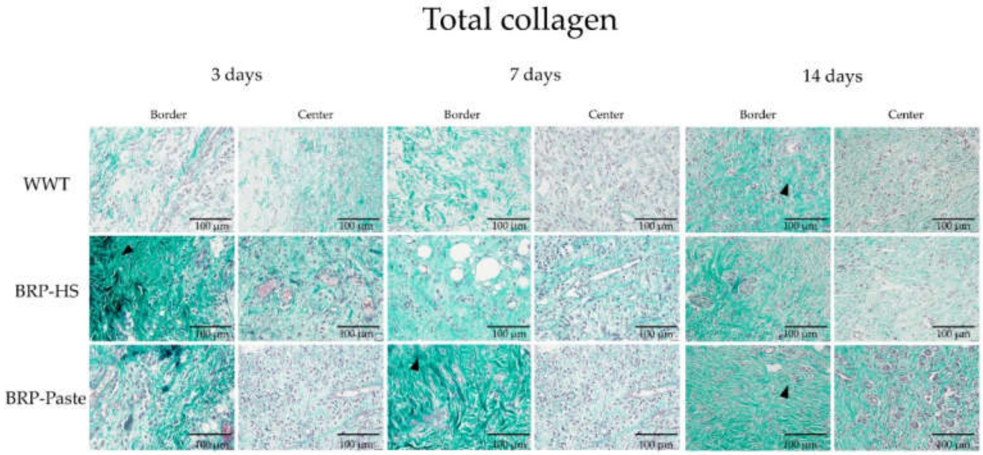

Figure S4. Photomicrographs of the dermis (40x), border and central regions of wounds from each experimental group in 3, 7 and 14 days of treatment for total collagen count stained in Mallory's trichrome. Arrowheads indicate collagen fibers. Bars 100 μm.

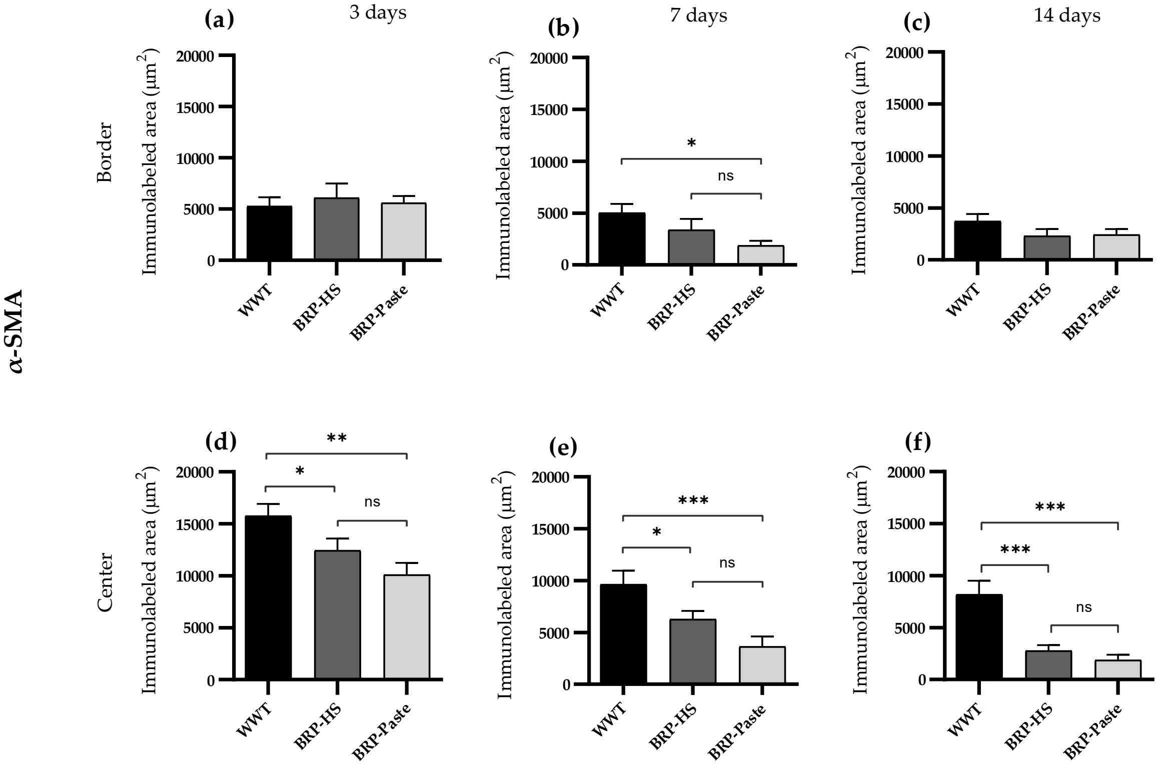

Figure S5. Quantification of α-SMA by area (μm^2) at border (a, b, c) and central (d, e, f) regions (dermis) of the wounds in each experimental group for the periods of 3 (a, d), 7 (b, e) and 14 (c, f) days of treatment. Statistical difference compared to the WWT group was obtained by one-way ANOVA followed by Newman-Keuls test in which $*p < 0.05$, $**p < 0.01$, and $***p < 0.001$ (n=6). ns = no significance.

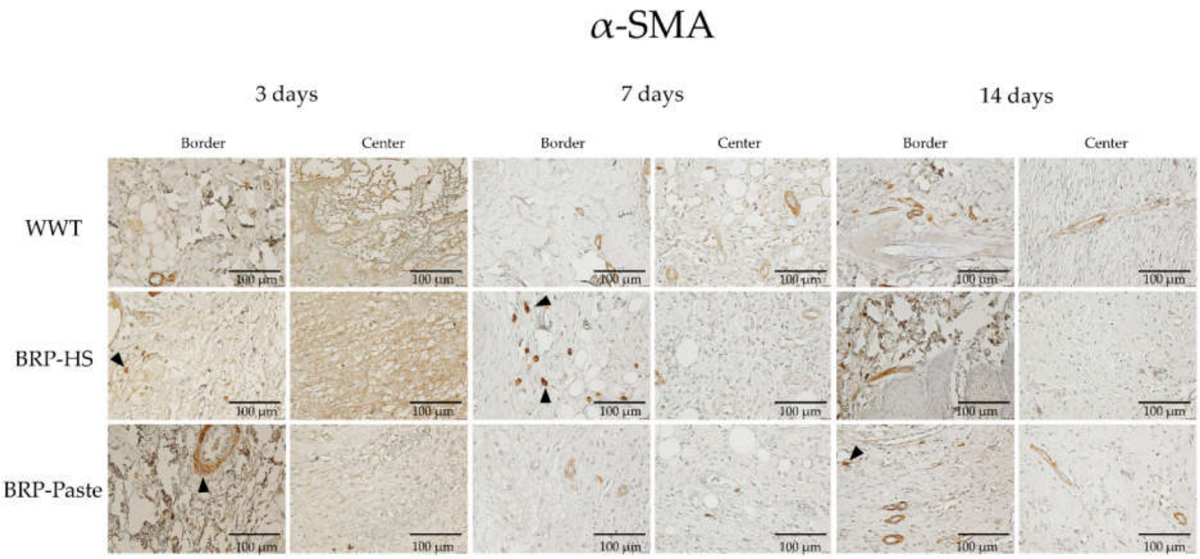

Figure S6. Photomicrographs of the dermis (40x), border and central regions of the wound from each experimental group in 3, 7 and 14 days of treatment for α-SMA immunolabeling. α-SMA positive cells represented by arrowheads are stained in brown. Bars 100 µm.

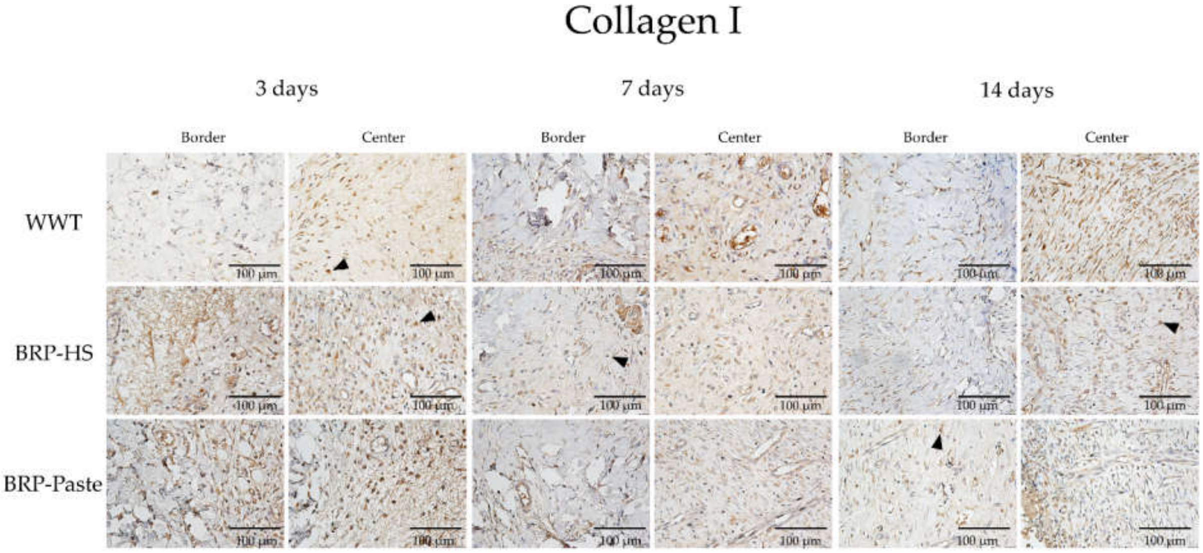

Figure S7. Photomicrographs of the dermis (40x), border and central regions of the wound from each experimental group in 3, 7 and 14 days of treatment for collagen type I immunolabeling. Collagen type I positive structures represented by arrowheads are stained in brown. Bars 100 µm.

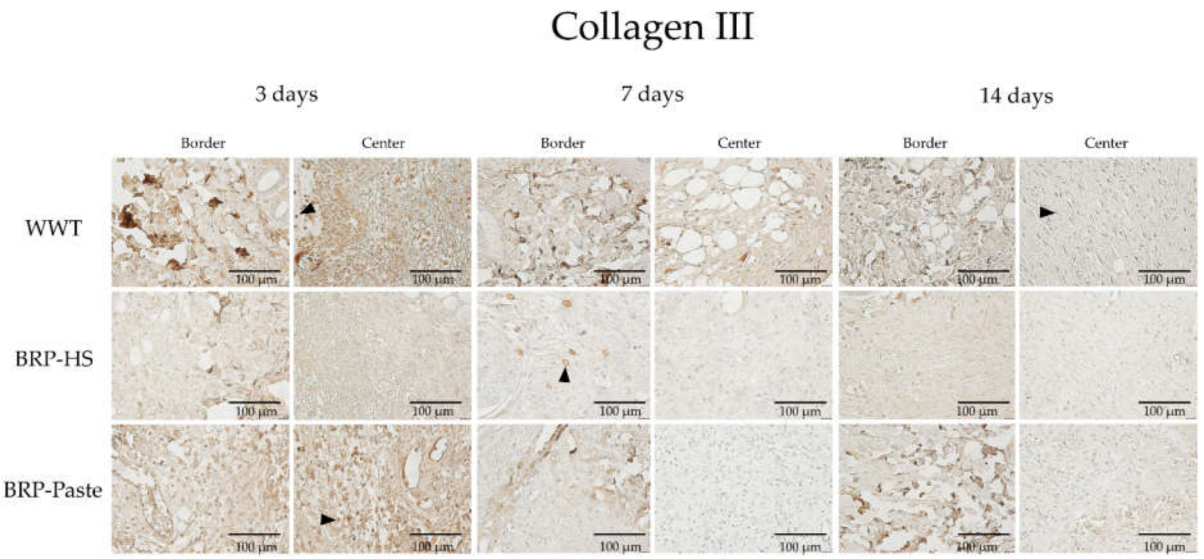

Figure S8. Photomicrographs of the dermis (40x), border and central regions of the wound from each experimental group in 3, 7 and 14 days of treatment for collagen type III immunolabeling. Collagen type III positive structures represented by arrowheads are stained in brown. Bars 100 µm.

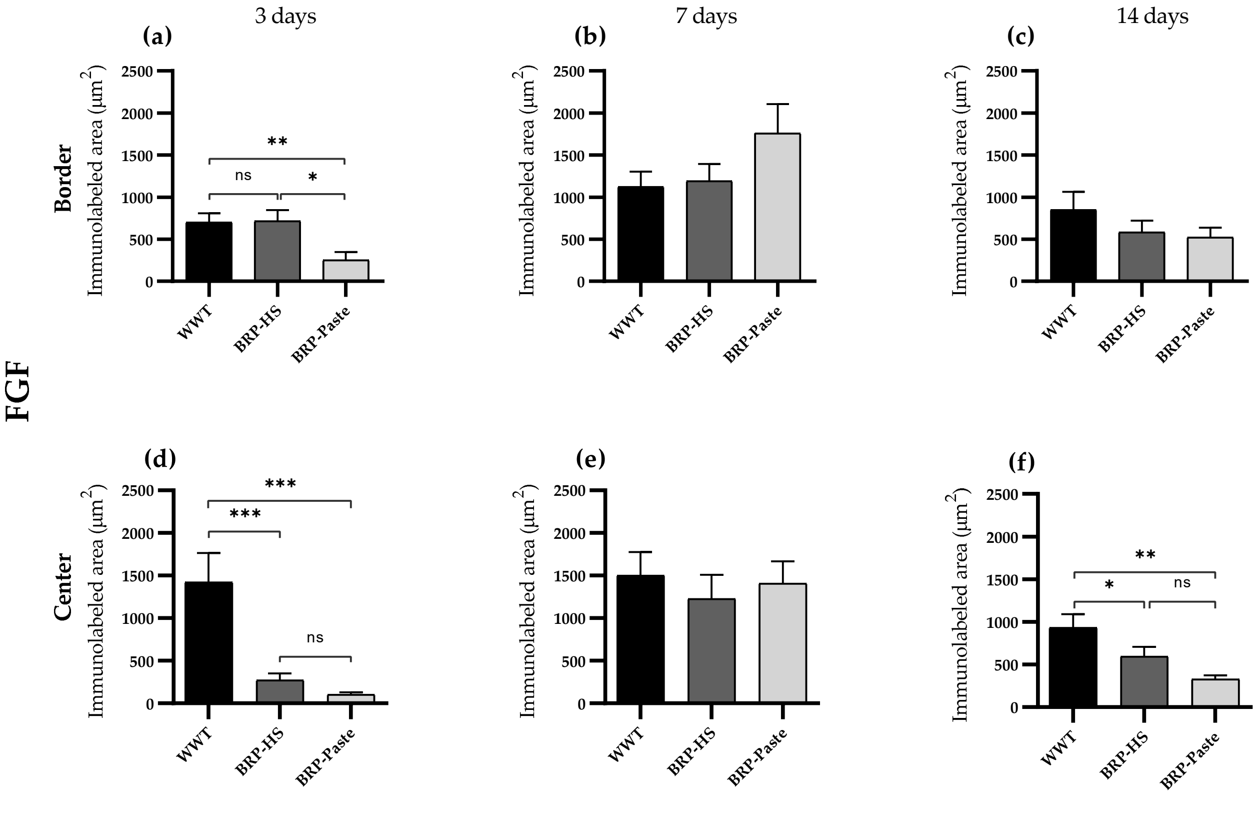

Figure S9. Quantification of FGF by area (µm²) at border (a, b, c) and central (d, e, f) regions (dermis) of the wounds in each experimental group for the periods of 3 (a, d), 7 (b, e) and 14 (c, f) days of treatment. Statistical difference compared to the WWT group was obtained by one-way ANOVA

followed by Newman-Keuls test in which $*p < 0.05$, $**p < 0.01$, and $***p < 0.001$ (n=6). ns = no significance.

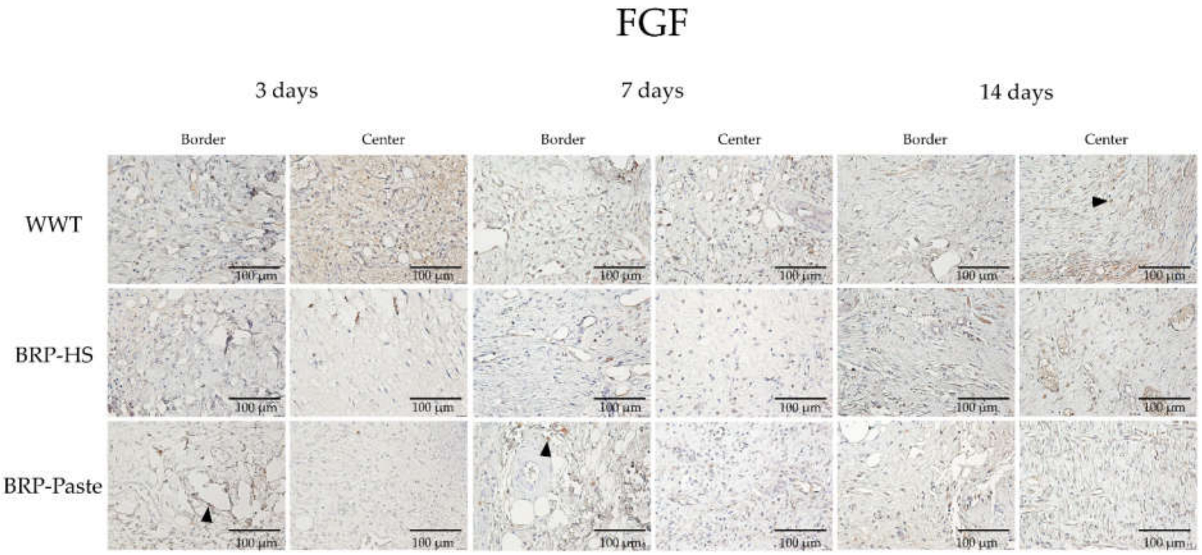

Figure S10. Photomicrographs of the dermis (40x), border and central regions of the wound from each experimental group in 3, 7 and 14 days of treatment for FGF immunolabeling. FGF positive cells represented by arrowheads are stained in brown. Bars 100 µm.

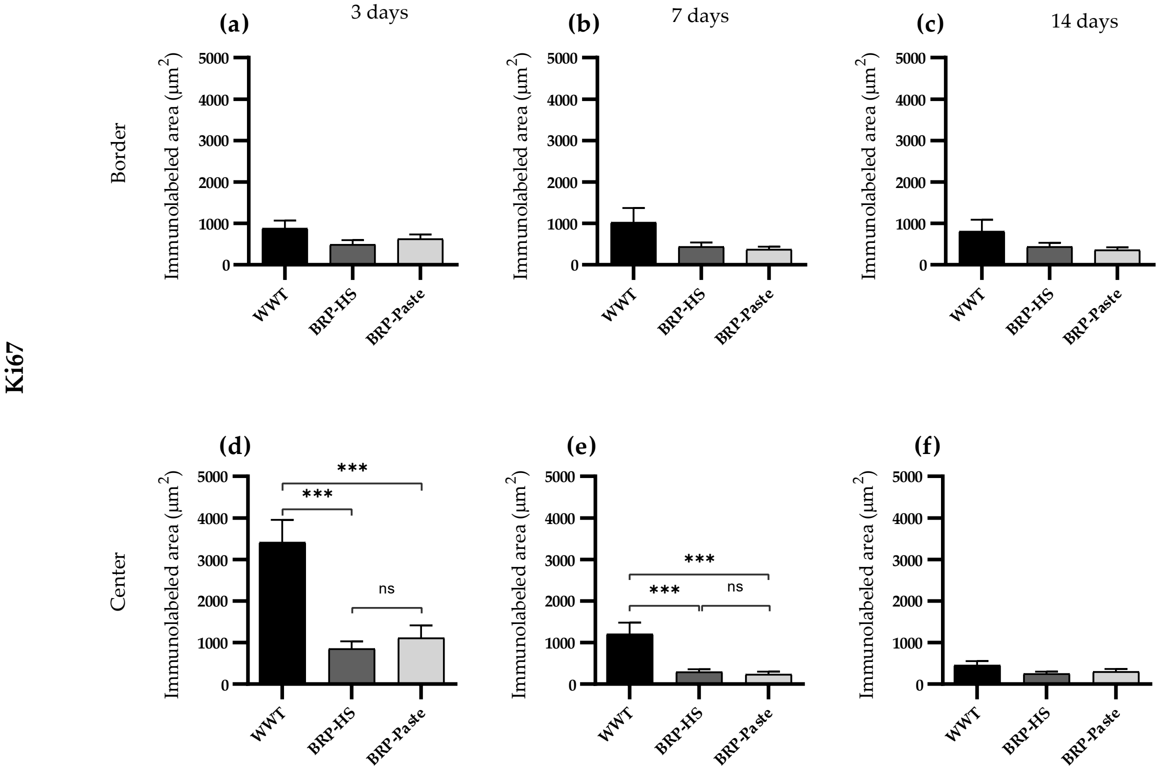

Figure S11. Quantification of Ki67 by area (µm^2) at border (a, b, c) and central (d, e, f) regions (dermis) of the wounds in each experimental group for the periods of 3 (a, d), 7 (b, e) and 14 (c, f) days

of treatment. Statistical difference compared to the WWT group was obtained by one-way ANOVA followed by Newman-Keuls test in which ***$p < 0.001$ (n=6). ns = no significance.

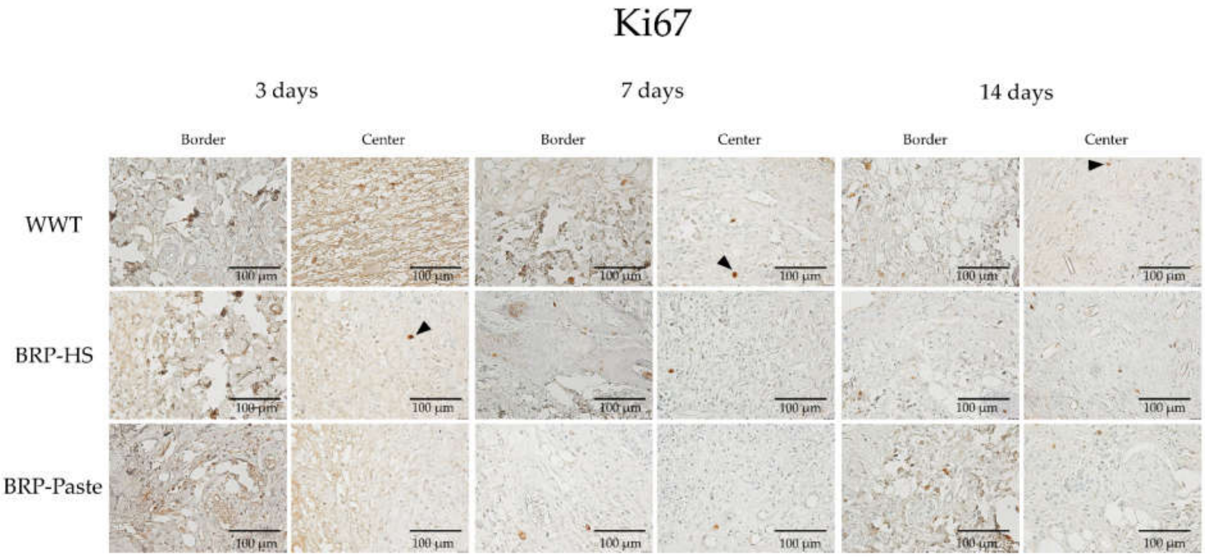

Figure S12. Photomicrographs of the dermis (40x), border and central regions of the wound from each experimental group in 3, 7 and 14 days of treatment for Ki67 immunolabeling. Ki67 positive cells represented by arrowheads are stained in brown. Bars 100 µm.

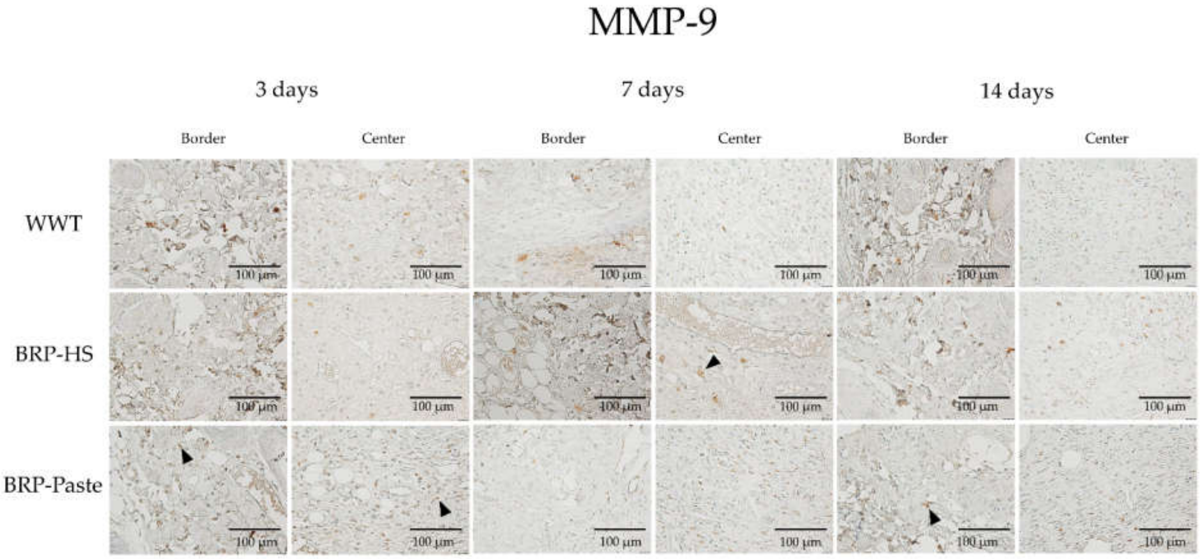

Figure S13. Photomicrographs of the dermis (40x), border and central regions of the wound from each experimental group in 3, 7 and 14 days of treatment for MMP-9 immunolabeling. MMP-9 positive cells represented by arrowheads are stained in brown. Bars 100 µm.

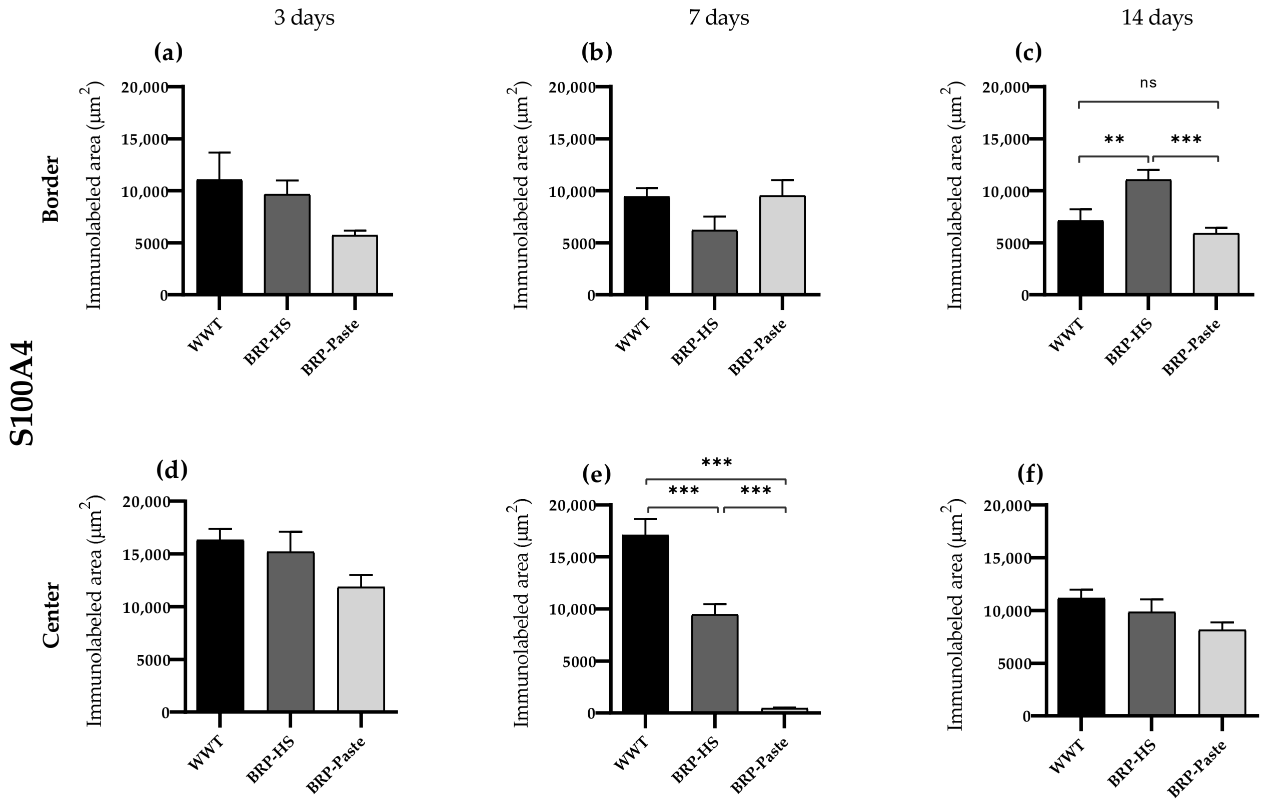

Figure S14. Quantification of S100A4 by area (μm²) at border (a, b, c) and central (d, e, f) regions (dermis) of the wounds in each experimental group for the periods of 3 (a, d), 7 (b, e) and 14 (c, f) days of treatment. Statistical difference compared to the WWT group was obtained by one-way ANOVA followed by Newman-Keuls test in which **p < 0.01, and ***p < 0.001 (n=6). ns = no significance.

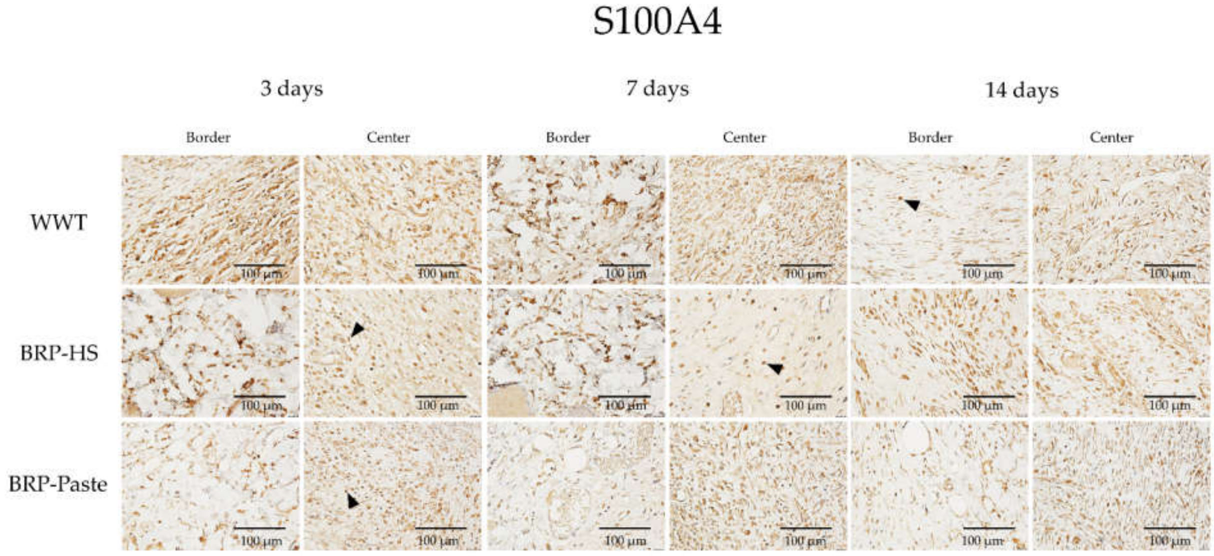

Figure S15. Photomicrographs of the dermis (40x), border and central regions of the wound from each experimental group in 3, 7 and 14 days of treatment for S100A4 immunolabeling. S100A4 positive cells represented by arrowheads are stained in brown. Bars 100 μm.

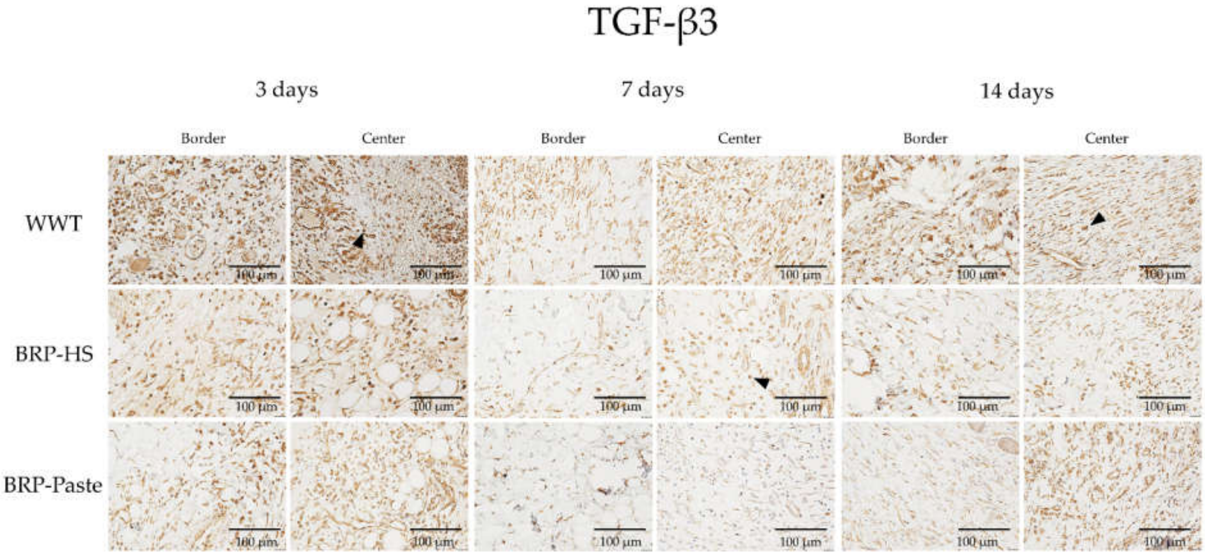

Figure S16. Photomicrographs of the dermis (40x), border and central regions of the wound from each experimental group in 3, 7 and 14 days of treatment for TGF-β3 immunolabeling. TGF-β3 positive cells represented by arrowheads are stained in brown. Bars 100 µm.
